# High Technology–Assisted Rehabilitation Based on Neuropsychological Assessments in a Case of Severe Acquired Brain Injury

**DOI:** 10.1155/crnm/5311669

**Published:** 2025-09-07

**Authors:** Cira Fundarò, Marina Maffoni, Mirella Boselli

**Affiliations:** ^1^Neuropathophysiology Unit of Montescano Institute, Istituti Clinici Scientifici Maugeri IRCCS, Montescano, Pavia, Italy; ^2^Psychology Unit of Montescano Institute, Istituti Clinici Scientifici Maugeri IRCCS, Montescano, Pavia, Italy; ^3^Neuromotor Rehabilitation Unit of Montescano Institute, Istituti Clinici Scientifici Maugeri IRCCS, Montescano, Pavia, Italy

**Keywords:** high technology–assisted rehabilitation, motor-cognitive rehabilitation, neuropsychological assessment, nontraumatic severe acquired brain injury (sABI), plasticity, upper limb rehabilitation

## Abstract

The rehabilitation of patients with severe acquired brain injury (sABI) presents various challenges. There is still a lack of knowledge regarding the efficacy and timing of high-technology (HT) rehabilitation in this clinical population. This paper describes the rehabilitation of a 56-year-old Caucasian woman who developed sABI due to the spontaneous rupture of multiple left middle cerebral artery aneurysms. The focus is on the interconnection between cognitive resources and motor-cognitive abilities to implement HT rehabilitation as early as possible, aiming to maximize the restoration of both motor and cognitive deficits. Following the acute clinical phase, the patient underwent an intensive multidisciplinary rehabilitation, which is described in this paper. The main target was the superior limb training with HT using an upper limb exoskeleton and augmented feedback exercises. The rehabilitative exercises have been selected and timed according to the neuropsychological assessment. The patient showed progressive cognitive and upper limb motor recovery along the tailored rehabilitative path. This case study provides useful insights into the value of a customized motor-cognitive HT rehabilitative approach, allowing for the best possible functional outcome in a case of sABI.

## 1. Introduction

Thanks to advances in medicine and science, clinical practice is increasingly asked to manage severe acquired brain injury (sABI) with different levels of severity [1]. Despite advances in acute stroke care, long-term motor impairments remain highly prevalent. While approximately 82% of patients regain independent ambulation, only 5%–34% recover full upper-limb function, underscoring the high prevalence of long-term motor impairments [2]. Moreover, subtle cognitive and motor deficits, often co-occurring, are commonly observed even after mild strokes and can significantly impact functional outcomes [3, 4]. These data underscore the necessity for continuous, multidimensional rehabilitation interventions.

Current rehabilitation strategies for these patients are particularly challenging as they are not unequivocally effective, depending on various characteristics (e.g., condition severity, age, and medical complications) [5, 6]. In the Italian context, the Salsomaggiore National Consensus Conference [6] and CICERONE Italian Consensus Conference [7] suggested a multidisciplinary care of neurological patients, including also high technology (HT) to treat acquired neurological disorders. Evidence supports the fact that early intensive and tailored rehabilitation interventions may maximize the possible outcome [8]. Indeed, there are various mechanisms by which exercise promotes recovery, such as enhancing synaptic plasticity, improving connections between brain hemispheres, and fostering neuronal regeneration [9, 10].

A key challenge lies in the dose of nonpharmacological interventions. Dose is a multidimensional construct, including intensity, frequency, and task complexity, that is often poorly reported and inconsistently defined in clinical research [11, 12]. Current rehabilitation practices are often limited in dose and intensity, despite converging evidence that high-dose, high-intensity therapy leads to significantly better outcomes compared to standard care [13]. The European Stroke Organisation recommends integrating at least 20 additional hours of repetitive upper-limb practice to enhance functional recovery [14]. Additionally, considerable inter-individual variability in recovery suggests that fixed-dose models may be insufficient [15]. Robotic upper-limb training further demonstrates that similar session durations can yield different outcomes depending on modality and movement quality [16]. Emerging technological solutions, including robotic-assisted and HT therapy, have the potential to bridge the gap of insufficient dose and intensity by enabling the delivery of high volumes of structured, task-specific repetitions, with studies showing positive correlations between training dose and functional improvements [9, 10, 17]. Preliminary evidence indeed confirms that technology-supported rehabilitation programs can feasibly increase daily therapy time and, thus, enhance overall treatment dose [17], overall accelerating the functional recovery [18]. Again, broader innovations and HT, including brain–computer interfaces, virtual reality, and neuromodulation, show promise in enhancing recovery by targeting neural plasticity, even in the chronic phase [19].

These advances highlight the urgent need for standardized, multidimensional frameworks to better articulate and individualize rehabilitation dose in the context of technology-supported recovery. To this scope, international guidelines for sABI also suggest considering a minimum core of clinical and functioning variables of the patient that can guarantee the effectiveness of a customized rehabilitation [20], while also paying attention to the patient's changes at each stage of the care path [21]. Indeed, the literature seems to underline the interconnection between cognitive profile (i.e., executive and attentional functions) and functional rehabilitation too [1, 22, 23]. In other words, cognitive resources may be a prerequisite for pursuing physical rehabilitation.

The most recent literature reported that robot-assisted and virtual reality rehabilitation may be a promising option for the rehabilitation of sABI [8, 16, 19, 24–26]. Specifically, the literature suggests that HT—understood as the integration of any advanced technological tools (e.g. robotics, virtual reality, etc.) into clinical practice to enhance, personalize, and optimize the patient's recovery—may foster brain plasticity, accelerate neurological damage recovery [9, 27], and improve cognitive profile too [9, 22, 28]. The literature supports the use of robotic or sensor-based tools for the simultaneous evaluation of sensorimotor and cognitive deficits [29]. This potential explains why HT may be a promising avenue for improving outcomes in patients with sABI. However, further studies and evidence are needed to assess the efficacy of these rehabilitation methods. Specifically, there is not yet a clear consensus on *how* (i.e., doses and approaches and physical and cognitive constraints) and *when* (i.e., the adequate time to take advantage as much as possible of neuroplasticity) to introduce different kinds of traditional and HT rehabilitation programs to maximize the motor-cognitive outcome. A consensus on considering clinical and cognitive variables over time may help to optimize the outcomes [30, 31].

Therefore, to understand whether a rehabilitation intervention with HT could be successfully proposed, we present a case of sABI due to subarachnoid hemorrhage and HT upper-limb rehabilitation, providing the core concepts according to CARE guidelines (CAse REport checklist) [32, 33]. Moreover, the case description was guided by the principles of the WHO International Classification of Health Interventions [34], keeping in mind the three core elements: *Target*, that is, the HT upper-limb rehabilitation of a patient with sABI, tailored to her evolving cognitive profile over time (Table 1); *Action*, describing a multidisciplinary, customized rehabilitation intervention; and *Means* intended as an integrated approach combining usual care with HT interventions. Therefore, this case aims to promote clinical reflections on the following question: Is there a specific time to maximize the rehabilitation outcome in complex clinical cases by continuously remodeling and customizing the interventions according to the patient's changing cognitive and functional levels?

## 2. Case Presentation

### 2.1. Brief Clinical Description

**Figure 1:**
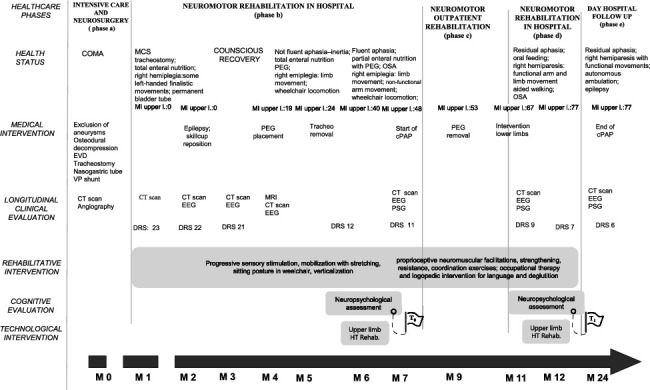
Summary of the timeline of patient care. Legend: EVD: external ventricular drain, CT scan: computed tomography scan, MRI: magnetic resonance imaging, MCS: minimally conscious state, EEG: electroencephalogram, PEG: percutaneous endoscopic gastrostomy, OSA: obstructive sleep apnea, cPAP: continuous positive airway pressure, PSG: polysomnography, VP shunt: ventriculoperitoneal shunt, DRS: Disability Rating Scale [41], MI upper l.: Motricity Index upper limb [42], and M: months.

The case presents a 56-year-old woman with subarachnoid hemorrhage without a previous history of disease. The patient developed sABI due to the spontaneous rupture of multiple left middle cerebral artery aneurysms with a left frontotemporal ischemic-hemorrhagic area.  Figure 1 visually summarizes the timeline of patient care.

Following the acute phase *(Figure 1—phase a: intensive care and neurosurgery),* the patient arrived in our hospital in the north of Italy. It is a specialized facility for intensive neurorehabilitation and it is equipped with a wide range of robotic devices, exoskeletons, and virtual reality platforms. These technologies are part of the center's broader strategic investment and are routinely used alongside traditional rehabilitation approaches when clinically appropriate. The ultimate scope is to utilize all available tools to optimize patient outcomes within a personalized, evidence-informed clinical framework. Here, the patient underwent a customized rehabilitation program both in the hospital and later in the outpatient regimen to maximize the restoration of motor and cognitive deficits. A multidisciplinary rehabilitation board, mainly comprising a neurologist, a physiatrist, and a neuropsychologist, was involved in designing a patient-tailored treatment plan and in defining the overall rehabilitation approach. This paper indeed focuses on the description of rehabilitation and the clinical process (Figure 1).

At the admission in our neurorehabilitation unit, 4 weeks after stroke *(Figure 1—phase b: neuromotor inpatient rehabilitation—*1^st^*admission)*, the patient's clinical neurological examination highlighted low vigilance (somnolence), rare spontaneous eye-opening, absence of language, right hemiplegia with hypertonic tone in the lower limb, and some left-handed finalistic movements. Passive mobilization revealed a slight joint limitation in the final degrees of shoulder abduction and flexion, as well as an incomplete fist closure (Table 2) [41, 42]. However, these impairments did not significantly affect functional use. In contrast, gait was notably limited due to the development of bilateral sural muscle retraction, which subsequently required surgical intervention. Hemiplegia was characterized by only a mild increase in muscle tone, which did not affect joint range of motion.

During the rehabilitation project *(Figure 1—phase c: neuromotor outpatient rehabilitation),* the patient showed a slow progressive neurological improvement, in terms of limb movement—from right hemiplegia, nonfunctional arm movement, and limb movement (M6)—and language—showing nonfluent aphasia (M6) and then fluent aphasia (M7).

One year after stroke *(Figure 1—phase d: neuromotor inpatient rehabilitation—*2^nd^*admission)*, the clinical reassessment at the end of the rehabilitation unveiled a global improvement of vigilance, motricity (arm and limb movement), articularity, and cognitive function.

At the last outpatient follow-up *(Figure 1—phase e: day hospital follow-up)*, 2 years after stroke, her motor and clinical conditions remained good and stable (residual aphasia, right hemiparesis with functional movements, and autonomous ambulation); it was possible to stop the use of the continuous positive airway pressure device. However, the persistence of seizure crises limited her functional independence.

### 2.2. Neuropsychological Assessment

To assess the changes in cognitive profile and customize the rehabilitation intervention based on cognitive reserve [44, 45], six months after the stroke, the patient underwent extensive neuropsychological assessments (see Table 1), which were conducted as early as possible based on the patient's cognitive functioning and level of disability as determined by the Disability Rating Scale (DRS) [41]. This allows us to unveil the minimal attentional and information-processing skills to introduce rehabilitation, as suggested in the literature [46, 47]. Overall, the first neuropsychological evaluation (*T*_0_*, Figure 1—phase b: neuromotor inpatient rehabilitation—*1^st^*admission)* unveiled specific deficits in selective attention tests and verbal memory measures probably linked to language disorders; 1 year later, neuropsychological assessment (*T*_1_*, Figure 1—phase d: neuromotor inpatient rehabilitation*—2^nd^*admission)* showed a significant improvement in previous impaired or low-end normal areas.

### 2.3. HT Augmented Feedback Exercises and Rehabilitative Intervention

Prior to initiating the rehabilitation (Figure 1—phase b: neuromotor inpatient rehabilitation—1^st^ admission), a neuropsychological evaluation (Table 1) was performed to identify the minimal cognitive prerequisites necessary for understanding and engaging in specific motor-cognitive tasks offered by the Armeo® Spring [43], a mechanical exoskeleton that replicates upper-limb joint movements simulating daily activities. This device is part of the rehabilitation equipment of our hospital and it was used to provide the patient with an upper limb-dedicated rehabilitation as it has been widely employed in such kind of neurorehabilitation [48, 49]. Specifically, based on the patient's neuropsychological profile—assessed through a comprehensive test battery [35–40, 50–53] at baseline (*T*_0_) and reassessment (*T*_1_)—we implemented a rehabilitation plan tailored to her evolving cognitive status (Table 3). Overall, two treatment cycles using Armeo® Spring [43] were administered at different stages of the rehabilitation path (M6 and M11-M12).

More in detail, the patient underwent right upper-limb HT therapy 5 days per week, with 30-minute sessions per arm, alongside conventional rehabilitative motor training (gait, posture, joint mobility, and muscle strengthening) 5 days per week for 60 min (Figure 1).

The structure of the HT rehabilitation was designed to enhance neuroplasticity through a task-oriented training based on three key principles: *focused* (i.e., goal-directed exercises), *intensive* (i.e., frequency and repetition with each session including several repetitions per exercise), and *progressive* (i.e., exercise difficulty levels were graded and adapted based on the patient's motor improvement, ranging from very easy to difficult).

Each Armeo® Spring [43] session typically comprised a combination of joint-specific and multijoint exercises. The patient's program included 3 single-joint tasks (n. 2 for the shoulder, n. 1 for the elbow) and 9 multijoint tasks involving various combinations of shoulder/elbow (n. 4), shoulder/wrist (n. 1), shoulder/grasping (n. 1), shoulder/elbow/wrist/grasping (n. 1), and shoulder/elbow/grasping (n. 1). Movements were distributed across vertical (n. 4 exercises) and horizontal (n. 3 exercises) planes, with five exercises involving multiplane motion. Additionally, the patient performed 2 reaching tasks, 2 grasping tasks, 12 spatial exploration tasks, and 3 cognitive-motor integration tasks.

Each exercise lasted approximately 2 min, and the platform provided real-time visual and auditory feedback (e.g., performance scores and target completion signals), enhancing motivation and engagement throughout the rehabilitation process. In addition, to enable active participation even in the presence of significant motor deficits, weight support mechanisms intrinsic to the device allowed for unloading of individual joints or of the entire upper limb. This feature was particularly important in the early stages of rehabilitation, enabling the patient to initiate training despite limited voluntary movement. Neither the range of motion limitations nor the slight hypertonia of our patient interfered with the feasibility of upper-limb HT treatment.

Therefore, the Armeo® Spring [43] protocol was employed not only to promote motor recovery [54] but also to stimulate cognitive processes involved in motor planning, visuospatial reasoning, and executive functioning [45, 55] (Table 2).

### 2.4. Training Results

The Armeo® Spring [43] device also offered a functional assessment to evaluate functional performance including vertical capture, horizontal capture, and reaction time. Specifically, the patient improved vertical capture in the first cycle and this betterment has been strengthened in the second cycle; horizontal capture partially and totally improved, respectively, in the first and second cycles; moreover, the patient significantly enhanced her reaction time in the first cycle and this outcome is maintained in the second one (Table 2). The rehabilitation outcomes go beyond Armeo® Spring scores [43]. Instead, they reflect quantitative movement and motor and cognitive parameters, consistent with the concept of precision rehabilitation enabled by sensor-based tracking and machine learning approaches [56]. We indeed carried on standard clinical assessments, including the Motricity Index (MI) [42] for motor function (Table 2) and a comprehensive cognitive evaluation (Table 3).

## 3. Discussion

The current case study describes HT rehabilitation based on neuropsychological assessments of sABI in a 56-year-old Caucasian woman, who experienced a subarachnoid hemorrhage caused by multiple middle cerebral artery aneurysms. In the current healthcare landscape, the rehabilitation interventions of patients with sABI are a significant challenge. Early rehabilitative interventions seem to be promising for reaching the best possible outcomes [6–8]. Thus, our patient underwent an ongoing HT rehabilitation program soon after the stabilization of the clinical status and the level of vigilance, reporting significant cognitive and motor improvements. What is replicable is not necessarily the use of a specific device, but rather the methodology, that is, the timely implementation of the intervention in relation to the patient's plasticity potential, and the selection of inclusion criteria based on preserved cognitive abilities. This rationale is grounded in neurophysiological principles suggesting that plasticity remains accessible in adults even after severe brain injury, provided that the intervention is appropriately timed and structured [57].

Firstly, the upper limb HT rehabilitation was offered based on the previous literature examples [48, 49, 58, 59]. However, controversy remains regarding the application of HT-assisted rehabilitation in patients with sABI. On the one hand, the literature stated that rehabilitation with technological devices may play a pivotal role in the treatment of upper-limb dysfunctions and in promoting motor and cognitive recovery [22, 23, 60, 61]. In addition, technological innovations offer promising means to address the current gap in rehabilitation intensity by facilitating the delivery of large volumes of structured, task-oriented practice. Initial evidence indicates that technology-enhanced rehabilitation programs can effectively extend daily therapy duration, thereby increasing the overall rehabilitation dose [17]. On the other hand, it has also been reported that motor-cognitive interventions with technological devices on the upper limb in subacute poststroke patients are at least equivalent to neurocognitive intervention without technological devices [62]. Probably, it is possible that the lack of consistency between results on the efficacy of HT rehabilitation may be at least partially related to some overstimulation effects [63, 64]. Beyond technical contraindications that may limit the use of specific devices—for example, the Armeo® Spring [43], which requires the absence of significant joint blocks—it is also crucial to consider the patient's cognitive level. Although these devices can enhance motivation and engagement and facilitate movement execution, a minimum level of consciousness and cognitive function is required for the patient to actively benefit from such facilitation. Otherwise, the intervention may become counterproductive, either due to lack of utility or excessive stimulation. In other words, it is necessary a consensus on *how, when*, and *why* to introduce HT interventions. In our opinion, the taking-care approach must be particularly patient-oriented [20, 44]. In this sense, we decided to propose a rehabilitation program using HT, the Armeo® Spring [43], on the basis of minimal cognitive prerequisites detected by neuropsychological assessments [45]. To date, physical and motor aspects are the main focus of rehabilitation strategies, with little attention paid to the patient's cognitive condition [65]. However, as evidence of the link between cognitive and motor functions grows, it is pivotal to establish rehabilitation programs that consider cognitive factors to customize the care path [44, 66]. We are convinced that the cognitive profile can be a kind of compass to determine the possible use and potential effectiveness of HT rehabilitation and, in the meantime, motor rehabilitation itself may increase cognitive performance too. Following this reasoning, in our opinion, it is mandatory to carry out neuropsychological assessments over time, especially in these kinds of patients, to define both the motor functions that may benefit from rehabilitation and the adequate timing to introduce it [63].

Another point of ongoing debate relates to the cost implications of using advanced rehabilitation technologies. A recent systematic review by Cano-de-la-Cuerda et al. [67] highlights the complexity and controversy surrounding the economic evaluation of robotic and virtual reality systems in neurological rehabilitation. The authors emphasize the substantial heterogeneity among studies in terms of devices, patient populations, protocols, and clinical settings, which limits the generalizability of current cost-effectiveness analyses. The key point is that the review suggests these technologies—though often perceived as costly—may actually contribute to cost optimization over time, particularly when evaluated from a long-term perspective. Increased hours of use may lead to a reduction in complications and therapist workload, potentially making these systems more cost-effective in the long run. Although further research is needed to establish the comparative benefits of such technologies over simpler and less expensive solutions, this case illustrates how, in well-equipped settings, technology can be meaningfully integrated into a personalized, multidisciplinary approach to rehabilitation.

Thus, the HT rehabilitation with an augmented feedback environment should be proposed to sABI, considering the damaged brain areas, as well as different perspectives, such as motor and cognitive (e.g., attention and executive functions) ones, to promote the best possible quality of life and the patient's psychophysical well-being.

Overall, this case report highlights the importance of a multidisciplinary and individualized approach in the management of such challenging patients. In addressing the timing of interventions, we aimed to suggest potential therapeutic windows that may optimize neuroplasticity and improve rehabilitative outcomes. In this context, the progression of the patient's cognitive profile was used as a clinical guide to tailor interventions and inform decision making in line with emerging evidence [1, 19, 22, 23]. Furthermore, this report contributes to the ongoing discussion regarding the integration, based on cognitive profile changes, of novel technologies into motor recovery strategies and the broader rehabilitation process for patients with sABI. Thus, motor and functional rehabilitation was delivered progressively, integrating advanced technologies—available as standard equipment in our rehabilitation gyms—as part of a broader, multidisciplinary strategy. However, as a single-case report, this study does not allow for causal inferences, and the findings are inherently descriptive and hypothesis-generating. While it does not follow a single case experimental design [68], it reflects a real-life clinical management scenario within a high-complexity neurorehabilitation setting. Thus, this paper does not intend to offer an exhaustive or universally applicable model; it seeks to provide a meaningful clinical example that may inspire further reflection and contribute to the refinement of clinical pathways in neurorehabilitation.

## Figures and Tables

**Table 1:** Association between cognitive tests and augmented feedback exercises.

Rehabilitation exercises (Armeo® Spring [34])	Cognitive prerequisite	Neuropsychological tests used
Remember instructions (across all exercises)	Verbal memory	Babcock short-tale [35]
		Rey 15-words, delayed recall [36]

Space exploration	Executive functions	Frontal assessment battery—FAB [37]
	Visual attention	Trail making test (part A) [38]

Follow trajectories to reach/grasp stimuli	Executive functions	Frontal assessment battery—FAB [37]
	Visual attention	Trail making test (part A) [38]
	Visual exploration	Trail making test (part A and part B) [38]
	Visual memory	Corsi span [39], Rey–Osterrieth complex figure—copy [40]

Reaction time	Executive functions	Frontal assessment battery—FAB [37]
	Attention	Trail making test (B and A) [38]

Avoid errors/confounding stimuli	Divided attention	Trail making test (part B) [38]

**Table 2:** Upper limb clinical and motor outcomes over time.

Armeo® Spring	First cycle (6 months after stroke)		Second cycle (1 year after stroke)	
a. Vertical capture	75% (1.11 115 s) –100% (1.27 101 s)		85% (1.28 78 s) –100% (1.14 43 s)	
b. Horizontal capture	44% (1.16 113 s) –94% (1.25 76 s)		72% (1.17 84 s) –100% (1.45 57 s)	
c. Reaction time	5% (60 s) –100% (216 s)		100% (248 s) –100% (172 s)	

**Motricity Index (MI) for the upper limb**				

**MI scores**	**First hospitalization**		**Second hospitalization**	
	**Pretreatment**	**Posttreatment**	**Pretreatment**	**Posttreatment**

MI pinch	11	19	22	26
MI elbow	14	14	25	25
MI shoulder	14	14	19	25
MI total	40	48	67	77

*Note:* a, b, c are measures provided by the Armeo® Spring device [43] concerning movements on the vertical plan (a), horizontal plan (b), and patient's reaction time (c). The measures are displayed in terms of achieved tasks percentage (%) and time (s = seconds); M: months pre and postrehabilitation.

**Table 3:** Neuropsychological assessments.

Tests (female, 9 years of schooling)	Range	NV	CS (ES) 6 months after stroke (*T*_0_)	CS (ES) 1 year after stroke (*T*_1_)
Mini mental state examination [50]	0–30	≥ 23.8	27.99 (corrected score)	/
Frontal assessment battery—FAB [37]	0–18	≥ 13.4	2	4
Trail making test (part A) [38]	/	< 94	1	4
Trail making test (part B) [38]	/	< 283	2	4
Digit span [39]	0–9	≥ 3.75	3	/
Corsi span [39]	0–10	≥ 3.75	4	/
Babcock short-tale [35]	0–28	≥ 7.5	1	4
Rey 15-words, immediate recall [36]	0–75	≥ 28.53	2	/
Rey 15-words, delayed recall [36]	0–15	≥ 4.69	4	/
Rey–Osterrieth complex figure—copy [40]	0–36	≥ 28.88	4	/
Rey–Osterrieth complex figure—recall/15” [40]	0–15	≥ 4.69	4	/
Test Giudizi Verbali (test verbal judgments) [51]	0–60	> 32	2	/
Raven SPM [52]	0–48	> 20.72	3	/

*Note:* NV = normative value; CS = corrected score; ES = equivalent score (ES 0-1 = defective/borderline performance, ES 2 = low-end normal performance, and ES 3 and 4: normal performance [53]). At *T*_0_, test showed specific deficits in selective attention tests and verbal memory measures. Overall, at *T*_1_, the neuropsychological assessment showed improvement in executive functions, selective and divided attention, and verbal memory.

## Data Availability

The authors declare that the clinical information presented in this case report is available from the corresponding author upon reasonable request.
